# Alkalosis in Critically Ill Patients with Severe Sepsis and Septic Shock

**DOI:** 10.1371/journal.pone.0168563

**Published:** 2017-01-03

**Authors:** Simon Kreü, Allan Jazrawi, Jan Miller, Amir Baigi, Michelle Chew

**Affiliations:** 1 Institute for Clinical Sciences Malmö, Lund University, Lund, Sweden; 2 Department of Cardiothoracic and Vascular Surgery, Örebro University Hospital, Örebro, Sweden; 3 Department of General Surgery, Västmanland County Hospital, Västerås, Sweden; 4 Institute of Medicine, General Medicine and Public Health, Gothenburg University, Gothenburg, Sweden; 5 Department of Anaesthesia and Intensive Care, Linköping University Hospital, Linköping, Sweden; Bambino Gesù Children's Hospital, ITALY

## Abstract

**Introduction:**

Although metabolic alkalosis is a common occurrence in intensive care units (ICUs), no study has evaluated its prevalence or outcomes in patients with severe sepsis or septic shock.

**Methods:**

This is a retrospective cohort study of critically ill patients suffering from severe sepsis and septic shock admitted to the ICUs of Halmstad and Varberg County hospitals. From 910 patient records, 627 patients met the inclusion criteria. We investigated the relationship between metabolic alkalosis and mortality. Further, we studied the relationship between metabolic alkalosis and ICU length of stay (LOS).

**Results:**

Metabolic alkalosis was associated with decreased 30-day and 12-month mortalities. This effect was however lost when a multivariate analysis was conducted, correcting for age, gender, pH on admission, base excess (BE) on admission, Simplified Acute Physiology Score III (SAPS III) and acute kidney injury (AKI). We then analyzed for any dose-response effect between the severity of metabolic alkalosis and mortality and found no relationship. Bivariate analysis showed that metabolic alkalosis had a significant effect on the length of ICU stay. When adjusting for age, sex, pH at admission, BE at admission, SAPS III and AKI in a multivariate analysis, metabolic alkalosis significantly contributed to prolonged ICU length of stay. In two separate sensitivity analyses pure metabolic alkalosis and late metabolic alkalosis (time of onset >48 hours) were the only significant predictor of increased ICU length of stay.

**Conclusion:**

Metabolic alkalosis did not have any effect on 30-day and 12-month mortalities after adjusting for age, sex, SAPS III-score, pH and BE on admission and AKI in a multivariate analysis. The presence of metabolic alkalosis was independently associated with an increased ICU length of stay.

## Introduction

Metabolic alkalosis is the most common acid-base disorder in intensive care patients [1]. Despite this there is only very limited research investigating the effects of metabolic alkalosis in this group of patients. It is often perceived as a relatively benign state and only life-threatening in extreme cases.

The effect of alkalosis has recently been demonstrated in a study investigating the association between serum (S-) bicarbonate levels and mortality in critically ill patients [2]. This study showed a U-shaped association between S-bicarbonate and mortality as well as ICU length of stay. Another recently published study investigated the associations of bicarbonate and acid-base status with mortality in healthy older individuals [3]. This study showed that metabolic alkalosis was associated with higer mortality.

Alkalosis is defined as an arterial pH exceeding the body’s normal pH (>7.45). Metabolic alkalosis is primarily due to increased losses of non-titratable acids resulting in an excess of HCO_3_^-^ or a decrease in H^+^ concentrations [4]. An arterial pH>7.45 and base excess (BE) >+3 mmol/L is generally classified as metabolic alkalosis.

In patients with sepsis and trauma metabolic alkalosis is most often a result of treatment given to correct hypotension, shock and acidosis. In these situations patients are often given large doses of citrated blood, Ringer’s acetate and sometimes bicarbonate [1]. In addition there may be gastrointestinal losses due to nasogastric emptying, vomiting, diuretics, diarrhea and antibiotic therapy. In addition a majority of patients suffer from a volume contraction which perpetuates the metabolic alkalosis, since this leads to an increased renal absorption of sodium via the renin-angiontensin-aldosterone axis. Bicarbonate absorption follows that of sodium and renal secretion is simultaneously decreased [5].

The physiological response to metabolic alkalosis is hypoventilation via a chemoreceptor-initiated inhibition of the respiratory centre. The aim of this response is to retain CO_2_ and increase PaCO_2_. After about 6 hours, the kidneys start to excrete HCO_3_^-^ and retain H^+4^. Metabolic alkalosis is often followed by a decrease in blood K^+^ concentration as extracellular K^+^ is exchanged with H^+^ as the body attempts to maintain electroneutrality [4]. This manifests clinically as muscle weakness, pain and spasms. The most dreaded consequence of hypokalemia is arrhythmias, including ventricular fibrillation. Another (often) less-considered effect of alkalosis is a decreased oxygen delivery to tissues as the oxyhemoglobin dissociation curve is shifted to the left. Alkalosis is also a potent vasoconstrictor, leading to further tissue hypoxia [5].

Severe sepsis and septic shock are characterized by inadequate tissue perfusion with metabolic acidosis as a result. Its treatment may inadvertently lead to an ‘overcorrection’ ie. an opposite condition with metabolic alkalosis instead. Alkalosis has been reported to be the most common acid-base disorder in hospitalized patients [6]. Newer evidence indicate that this is also common in the critically ill [7].

The incidence of metabolic alkalosis has surprisingly not been documented in patients with severe sepsis and septic shock. Severely septic patients are at high risk of developing metabolic alkalosis during their ICU stay, and in these patients it seems logical to avoid an acid-base disorder which can potentially lead to even worse tissue perfusion.

In this study we aim to investigate the relationship between metabolic alkalosis and mortality, the latter defined as short-term (30-day) and long-term (12-month). We aimed also investigate the relationship between metabolic alkalosis and ICU length of stay. Our hypothesis is that metabolic alkalosis is associated with increased short- and long-term mortalities, as well as ICU length of stay.

## Materials and Method

### Design

This is a retrospective cohort study of patients with severe sepsis and septic shock admitted to the intensive care units of Halmstad and Varberg county hospitals.

### Ethics

This study was approved by the Regional Ethical Committee of Lund, Sweden (Dnr. 2014/923). The Ethical committee judged that informed consent was unnecessary due to the retrospective, non-invasive and ‘audit’ nature of the study. The study was conducted in accordance to the Swedish Data Protection Act.

### Patients

All patients >18 years of age admitted to the ICUs of Halmstad and Varberg county hospitals between 1 January 2008 and 31 December 2014 were identified via the Swedish Intensive Care Registry. The Swedish Intensive Care Registry (SIR) is a medical quality register which audits and benchmarks Swedish intensive care (The Swedish Intensive Care Registry. [cited 2015, 20th of November]. Available from: http://www.icuregswe.org.). SIR has prospectively collected data since 2001, and in 2015, does so from 77 of the 84 (92%) ICUs in Sweden. Data reported to SIR include details on individual patients’ diagnoses, SAPS III score, ICU length of stay (LOS) and diagnoses assigned during the ICU stay. Furthermore, SIR collects data on ICU outcome, and performs a prospective follow-up on vital status. Collected data is validated internally, and any identified inconsistencies or logical defects are returned to the local, submitting ICU for correction.

Patients with the diagnoses severe sepsis and/or septic shock (ICD codes A41.9, R65.1, R57.2) were included in this study.

### Materials

We retrieved data on arterial blood gas results from the Department of Clinical Chemistry, Halland county hospitals (covering both Halmstad and Varberg). All results for all arterial blood gas samples taken during ICU stay were included. We extracted data regarding age, gender, mortality, ICU stay, SAPS III-score and acute kidney injury (AKI) from SIR.

### Measurements and methods

Metabolic alkalosis was defined as BE>+3 and pH exceeding 7.45 at any time during ICU stay. The severity of alkalosis was defined as;”mild metabolic alkalosis” (pH = 7.45–7.50),”moderate metabolic alkalosis” (pH = 7.50–7.55), "severe metabolic alkalosis” (pH >7.55).

We chose to use BE as a relatively quick method of identifying whether or not the acid-base disorder had a metabolic component, as opposed to Stewart’s approach[8] which would have been more comprehensive but unreasonably time-consuming. Patients with BE>+3 but with pH = <7.45 were defined as ‘not alkalotic’. Patients with BE <+3 but pH greater than 7.45 were defined as ‘not metabolic alkalotic’. Respiratory alkalosis was defined as pH >7.45 and pCO_2_ <4.5 kPa. Patients with both respiratory and metabolic alkalosis were registered as having mixed alkalosis. Time of onset of metabolic alkalosis was registered. “Early onset” or “Late onset” metabolic alkalosis was defined as occurring within or after 48 hours of ICU admission respectively.

Patients in ICU often have multiple arterial blood gas samples taken throughout their ICU stay. We chose a priori to use the blood gas sample with the highest pH ad BE values. We also collected data regarding pH and BE on admission

### Statistics

No sample size calculation was made since there were no previous studies available to inform us on the incidence and mortality rates related to metabolic alkalosis. All data was analysed using Statistical Package for the Social Sciences (SPSS) version 23 (SPSS Inc, Chicago, Illinois, USA).

We analysed the association between metabolic alkalosis and mortality using the Chi-squared and Mann-Whitney U-tests. Further we explored this association using multivariate analysis, adjusting for potential confounders such as age, gender, SAPS III-score, pH and BE on admission and AKI. These were chosen a priori since they were clinically plausible confounders. We also investigated if there was a dose-dependent effect on mortality due to metabolic alkalosis.

The association between ICU length of stay and metabolic alkalosis was tested using a Mann-Whitney test. We also dichotomized ICU length of stay and analyzed for this using cross-tables and a Chi-squared test. The relationship was further explored in a multivariate analysis, adjusting for the same factors as above.

The association between early/late onset of metabolic alkalosis and all outcome parameters (30-day and 12-month mortalities, ICU length of stay) was tested using Chi-squared tests. Early metabolic alkalosis was defined as time of onset within 48 hours of ICU admission and late metabolic alkalosis was defined as time of onset after 48 hours of ICU admission. Similarly we explored the effect of mixed alkalosis on all outcome parameters. Mixed alkalosis was defined as pH > 7.45 together with BE>+3 and pCO_2_ <4.5 kPa. To assess the contribution of mixed and early/late alkalosis, we specified two separate sensitivity analyses in the multivariable model. In the first sensitivity analysis, we excluded all the patients with early onset metabolic alkalosis, and in the second, we excluded patients with mixed alkalosis.

A p-value <0.05 was considered significant.

## Results

### Population

**Fig 1:**
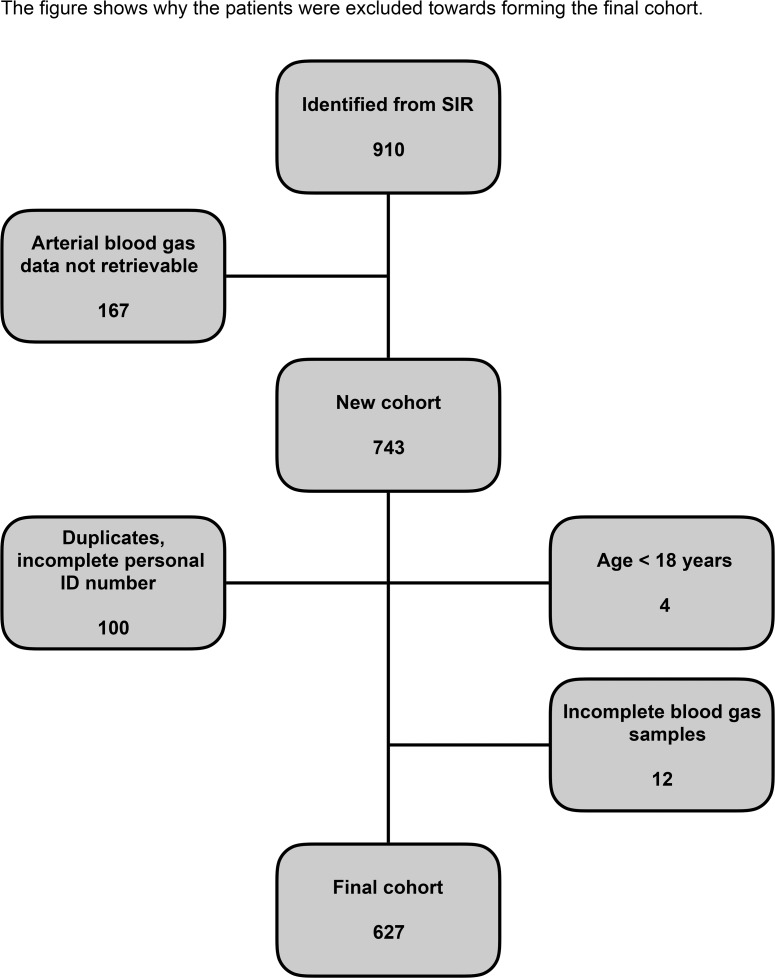
Flow chart of study population selection.

Nine-hundred and ten patients were identified with severe sepsis and septic shock from Halmstad and Varberg county hospitals between 1 January 2008 and 31 December 2014. Due to a change in the Department of Clinical Chemistry’s database, there was no available data from 5 October 2013 (167 patients). A further 116 patients were excluded due to missing or incomplete data (eg. no personal identification numbers, incomplete personal identifications numbers, no or incomplete blood gas data available in database, 4 patients < 18 years old). The final cohort consisted of 627 patients (Fig 1). The mean age of the population was 69 ±14 years. Mean ICU LOS was 5.4 ±6.6 days. 30-day mortality was 32.4% (203 patients), 12-month mortality 40.8% (256 patients). Two hundred and sixty six patients (42.4%) were diagnosed with metabolic alkalosis at any time during their ICU stay and 361 patients were non alkalotic. Other population characteristics are shown in Table 1.

**Table 1:** Baseline characteristics

	Total patients (*n* = 627)
Age (years) mean ± SD	69.0 ±14.1
Male [*n* (%)]	254 (40.5)
Hospital Halmstad [*n* (%)]	378 (60.3)
Hospital Varberg [*n* (%)]	248 (39.6)
ICU-LOS (days) mean ± SD	5.39 ± 6.56
Metabolic alkalosis [*n* (%)]	266 (42.4)
Mild metabolic alkalosis [*n* (%)]	104 (16.6)
Moderate metabolic alkalosis [*n* (%)]	126 (20.1)
Severe metabolic alkalosis [*n* (%)]	37 (5.9)
30 d mortality [*n* (%)]	203 (32.4)
12 m mortality [*n* (%)]	256 (40.8)
pH at admission (value) mean ± SD	7.36 ± 0.11
BE at admission (mEq/L) mean ± SD	-2.8 ± 6.0
SAPS III score (value) mean ± SD	67.89 ± 13.34

### Mortality

A bivariate analysis correcting for age demonstrated a protective effect of metabolic alkalosis, both for 30-day (p = 0.001) and 12-month (p = 0.011) mortality (Table 2). This effect was however lost when a multivariate analysis was conducted, correcting for age, gender, pH on admission, BE on admission and SAPS III-score and AKI. In this analysis the most important determinants for 30-day and 12-month mortality were age and SAPS III-score (Table 3). We then analyzed for any dose-response effect between the severity of metabolic alkalosis and mortality and found no relationship.

**Table 2:** Association between metabolic alkalosis and 30-day respectively 12-month mortality.

	30-day mortality			12-month mortality		
	No	Yes	Total	No	Yes	Total
**No metabolic alkalosis**	22562.3%	13637.7%	361100.0%	19854.8%	16345.2%	361100.0%
**Metabolic alkalosis**	19974.8%	6725.2%	266100.0%	17365.0%	9335.0%	266100.0%
**Total**	42467.6%	20332.4%	627100.0%	37159.2%	25640.8%	627100.0%

Pearson Chi-Square test for 30 day mortality, exact sig. (2-sided) *p* = 0.001

Pearson Chi-Square test for 12 month mortality, exact sig. (2-sided) *p* = 0.011

**Table 3:** Multivariate analysis on short-term and long-term mortality.

	30-day mortality				12-month mortality			
	OR	*p*-value	95% C.I		OR	*p*-value	95% C.I	
			Lower	Upper			Lower	Upper
**Age**	1.029	0.001	1.011	1.048	1.030	<0.001	1.013	1.046
**Female**	1.049	0.809	0.713	1.544	0.678	0.037	0.470	0.977
**Metabolic alkalosis**	0.691	0.075	0.461	1.037	0.819	0.302	0.560	1.197
**pH at admission**	0.282	0.293	0.027	2.980	0.333	0.339	0.035	3.172
**BE at admission**	0.965	0.134	0.921	1.011	0.979	0.339	0.936	1.023
**SAPS III**	1.058	<0.001	1.040	1.076	1.052	<0.001	1.035	1.069
**AKI**	1.163	0.775	0.714	1.895	1.275	0.785	0.799	2.034

### ICU length of stay

Patients with metabolic alkalosis had significantly longer ICU LOS than patients with no alkalosis (6 [3–10] vs. 2[1–4] days, p<0.001). We checked this result by dichotomizing around median ICU stay (3 days). Also here we showed a higher proportion of patients with metabolic alkalosis had ICU stays >3 days (p<0.001) (Table 4).

**Table 4:** Association between metabolic alkalosis and ICU length of stay.

	ICU length of stay		Total
	< = 3	>3	
**No metabolic alkalosis**	263 (73.1%)	97 (26.9%)	361 (100%)
**Metabolic alkalosis**	85 (32%)	181 (68%)	266 (100%)
**Total**	349 (55.7%)	278 (44.3%)	627 (100%)

Pearson Chi-Square, exact sig. (2-sided) *p*<0.001

We further explored this relationship in a multivariate analysis adjusting for age, gender, SAPS III-score, AKI, pH and BE at admission. This was significant—patients with metabolic alkalosis had increased ICU LOS with an odds ratio (OR) 6.461 (95% CI 4.421–9.443, p<0.001). When we investigated for a dose-response relationship between the severity of metabolic alkalosis and ICU LOS we found no clear linear relationship (Table 5). We also found a significant association between late alkalosis and ICU LOS (p<0.001).

**Table 5:** Association between metabolic alkalosis (any severity, mild, moderate and severe) with ICU length of stay.

	OR	*p*-value	95% C.I	
			Lower	Upper
**Age**	0.995	0.454	0.981	1.008
**Female**	1.078	0.678	0.755	1.541
**Metabolic alkalosis**	6.461	<0.001	4.421	9.443
**pH at admission**	1.654	0.656	0.181	15.120
**BE at admission**	0.965	0.115	0.924	1.009
**SAPS III**	1.002	0.751	0.988	1.018
**AKI**	1.468	0.107	0.920	2.341
Mild metabolic alkalosis*	2.256	<0.001	1.462	3.480
Moderate metabolic alkalosis*	4.485	<0.001	2.905	6.924
Severe Metabolic alkalosis*	2.678	0.007	1.317	5.444

*Separate analysis only adjusted for age.

In a sensitivity analysis excluding patients with mixed alkalosis, we found similar results to the general model. ‘Pure’ metabolic alkalosis was significantly associated with increased ICU LOS with an OR 5.171 (95% CI 3.454–7.541, p<0.001). Similarly, when exluding patients with early alkalosis, the occurence of metabolic alkalosis became the only significant predictor of ICU LOS (OR 9.04, 95% CI 6.02–13.57, p<0.001) (Table 6).

**Table 6:** Sensitivity analyses within the multivariate model on ICU LOS excluding mixed alkalosis and early onset alkalosis.

	Mixed alkalosis excluded				Early onset excluded			
	OR	*p*-value	95% C.I		OR	*p*-value	95% C.I	
			Lower	Upper			Lower	Upper
**Age**	0.991	0.190	0.978	1.004	0.991	0.182	0.977	1.004
**Female**	1.069	0.709	0.753	1.517	0.754	0.650	0.754	1.573
**Metabolic alkalosis**	5.171	<0.001	3.545	7.541	9.037	<0.001	6.019	13.569
**pH at admission**	2.609	0.393	0.289	23.589	2.342	0.467	0.236	23.212
**BE at admission**	0.975	0.253	0.934	1.018	0.975	0.266	0.933	1.019
**SAPS III**	1.006	0.400	0.992	1.021	1.003	0.722	0.987	1.018
**AKI**	1.361	0.185	0.863	2.149	1.314	0.266	0.813	2.125

## Discussion

In this cohort of 627 consecutive patients with severe sepsis and septic shock, we found that metabolic alkalosis occurring at any time during ICU stay was associated with increased ICU LOS. Contrary to our hypothesis, we could not demonstrate a deleterious effect of metabolic alkalosis on 30-day and 12-month mortality, after adjusting for the previously mentioned factors.

With regard to ICU LOS the association between metabolic alkalosis was observed regardless of its severity. Metabolic alkalosis was an independent predictive factor after adjustment for age, gender, SAPS III-score, AKI, pH and BE at admission. This was confirmed in 2 sensitivity analyses, where 1) patients with mixed respiratory and metabolic alkalosis were excluded, and 2) patients with early metabolic alkalosis were excluded. Indeed, the occurrence of metabolic alkalosis was the only predictive factor of increased ICU LOS with odds ratios between 5 and 9, regardless of how the data was analyzed, speaking for the robustness of these findings. The only other study shedding light on the effects of alkalosis in the critically ill is Liborio et al^2^. Our study differs in so far as we chose to specifically investigate patients with severe sepsis and septic shock. The clearly significant relationship between ICU length of stay and metabolic alkalosis deserves attention, however it is impossible to determine cause and effect in this study–does metabolic alkalosis confer an increased risk of prolonged ICU stay or vice versa? Increased time in the ICU exposes patients to interventions that may predispose to alkalosis. Indeed, the increase in odds ratio from 6.46 in the general model, to 9.04 when early alkalosis was excluded, supports the relative importance of late alkalosis. Regardless, these results are interesting at least from a socio-economic cost perspective, since intensive care is costly and may confer added risks to the patient if unnecessarily prolonged.

Contrary to Liborio et al^2^ we found a ‘protective’ effect of metabolic alkalosis on mortality. We believe that this is due to the fact that when dichotomizing data to patients with and without metabolic alkalosis we missed important subgroup differences. For example patients who were classified as ‘no metabolic alkalosis’ may in fact have been acidotic, a known risk factor for mortality. This is supported by data from our multivariate analysis, showing that metabolic alkalosis in itself was no longer a significant predictor of mortality. Instead, age and SAPS III-score were identified as the most important variables. We therefore speculate if acidosis may have affected survival in the non-metabolic alkalosis group. This hypothesis would be in line with Liborio’s finding of a U-shaped mortality curve where the extremes of S-bicarbonate adversely affect survival. In order to investigate this further one would have to further analyse the data in subgroups, dividing patients into severe acidosis, moderate acidosis, mild acidosis, severe alkalosis, moderate alkalosis, mild alkalosis. This was considered at the outset of our study, however, we realized that it was simply not dimensioned to analyze 6 subgroups.

Several limitations must be noted in this study. Firstly, the retrospective design precludes any conclusions regarding cause and effect, and the study must be seen as hypothesis-generating only. We have corrected for a number of potential confounders such as age, gender, SAPS III-score, AKI, pH and BE at admission, these were mostly identified a priori as ‘clinically plausible’. There are almost certainly a number of hidden confounders. Two such important variables that we would have liked to explore are the time spent on mechanical ventilation and the impact of renal replacement therapy.

A further limitation may be the fact that the study period was nearly six years long. We can therefore not exclude that changes in treatment during this period have a potential confounding effect on the outcome. Additionally our cohort came from two different, albeit, comparable regional hospitals. Local guidelines and treatment practices may have existed although it is our experience that these hospitals work closely together and have generally similar treatment guidelines. Notwithstanding, the limited catchment area and type of hospital enrolled has obvious implications for the generalizability of the findings. It should be noted that the old definitions of severe sepsis and septic shock were used since the study was conducted from 2008–2014 and written during 2015 predating the latest (2016) definitions.

Finally, the sample size here only allowed us to test a limited hypothesis. A larger sample size would have allowed stratification into more subgroups depending on the acid-base disorder.

In conclusion, our data demonstrate a significant association between the presence of metabolic alkalosis and ICU length of stay. This effect was present even after adjusting for potential confounders and was the only predictive factor demonstrated in two sensitivity analyses where patients with mixed alkalosis and early onset alkalosis were excluded. In contrast, and contrary to our hypothesis, metabolic alkalosis was not independently associated with mortality. Although metabolic alkalosis may not cause death, it is a condition that should be taken seriously since it is associated with negative patient outcomes. Further studies should investigate the effects of metabolic alkalosis on electrolyte homeostasis, renal function, cardiac and other complications as potential causes of the increased ICU length of stay.

## Supporting Information

S1 File Anonymous Datasheet. (XLSX)
