# Correction: Alkalosis in Critically Ill Patients with Severe Sepsis and Septic Shock

**DOI:** 10.1371/journal.pone.0175530

**Published:** 2017-04-05

**Authors:** The PLOS ONE Staff

There is an error in Table 2. The numbers and percentages have been merged together. Instead of reading 225 in the first row, first column, and the corresponding percentage 62.3% in the second row, first column, each cell reads 22562.3%. Please view the correct table below. The publisher apologizes for the error.

**Table 2:** Association between metabolic alkalosis and 30-day respectively 12-month mortality.

	30-day mortality			12-month mortality		
	No	Yes	Total	No	Yes	Total
**No metabolic alkalosis**	225	136	361	198	163	361
**No metabolic alkalosis (%)**	62.3%	37.7%	100.0%	54.8%	45.2%	100.0%
**Metabolic alkalosis**	199	67	266	173	93	266
**Metabolic alkalosis (%)**	74.8%	25.2%	100.0%	65.0%	35.0%	100.0%
**Total**	424	203	627	371	256	627
**Total (%)**	67.6%	32.4%	100.0%	59.2%	40.8%	100.0%

Pearson Chi-Square test for 30 day mortality, exact sig. (2-sided) *p* = 0.001

Pearson Chi-Square test for 12 month mortality, exact sig. (2-sided) *p* = 0.011
